# Activated autophagy of innate immune cells during the early stages of major trauma

**DOI:** 10.3389/fimmu.2022.1090358

**Published:** 2023-01-12

**Authors:** Deng Chen, Cong Zhang, Jialiu Luo, Hai Deng, Jingzhi Yang, Shunyao Chen, Peidong Zhang, Liming Dong, Teding Chang, Zhao-hui Tang

**Affiliations:** Department of Traumatic Surgery, Tongji Trauma Center, Tongji Hospital, Tongji Medical College, Huazhong University of Science and Technology, Wuhan, China

**Keywords:** autophagy, innate immune cells, major trauma, trauma-induced immune dysfunction, single-cell sequencing, monocyte, neutrophil

## Abstract

**Background:**

Trauma-induced immune dysfunction has been a major barrier to achieving reduced mortality, which is poorly understood. Autophagy is a crucial catabolic mechanism of immune cells during times of stress. Few studies have investigated the immune regulatory effects induced by autophagy after trauma. Here, we use single-cell transcriptomics analysis in a major trauma cohort to demonstrate the dominant role of autophagy in innate immune cells during the early stages of major trauma.

**Method:**

Single-cell transcriptional profiling of peripheral blood mononuclear cells (PBMCs) was performed, which were sampled from three control participants and five major trauma patients within 6 hours of injury. In detail, after single-cell RNA-sequence data processing, cell type annotation and cluster marker identification were performed. A genetic toolbox with 604 autophagy-related genes was used to monitor the autophagy levels in immune cells. In addition, all transcriptome RNA sequencing data obtained from PBMCs in a cohort of 167 major trauma patients were downloaded from gene expression omnibus (GEO) datasets (GSE36809). Key deregulated biological processes and important autophagic hub genes involved in immune cells were identified by weighted gene co-expression network analysis and gene ontology enrichment analysis.

**Results:**

A total of 20,445 differentially expressed genes were identified and five co-expression modules were constructed. Enrichment analysis indicated that activated autophagy is the most important biological process during the early stages of major trauma, and JMY (autophagy-related genes) were identified as hub genes. The single-cell transcriptional profiling of PBMCs demonstrated that all components of adaptive immune cells were significantly decreased, whereas components of innate immune cells (monocytes and neutrophils) were significantly increased in major trauma patients compared with control participants. Activated autophagy was detected in monocytes and neutrophils by monitoring the dynamic transcriptional signature of the autophagy-related genetic toolbox. Biological process analysis shows that antigen uptake, processing presentation, and major histocompatibility complex (MHC) class II protein complex assembly pathways were up-regulated in autophagy-positive monocytes, whereas antigen processing and presentation of endogenous antigen and type I interferon signaling pathways were up-regulated in autophagy-positive neutrophils during the early stages of major trauma.

**Conclusion:**

Our study demonstrated that autophagy is a biological process crucial to the development of immune disorders in the early stages of major trauma. Furthermore, the results of our study generated a comprehensive single-cell immune landscape for major trauma patients, in which we determined that autophagy profoundly affects the main functions of innate immune cells and provides insight into the cellular basis of immune dysregulation after major trauma.

## 1 Introduction

Trauma is a leading cause of global mortality and accounts for 10.1% of the global burden of disease. Annually, nearly 4.8 million people die from trauma-related injuries (1–3). Major trauma accounts for 3%–5% of total trauma incidents and is characterized by serious complications and a higher mortality rate, primarily because of fatal damage and unmanageable complications (4, 5). Hemorrhagic shock and overwhelming injury to vital organs are responsible for early mortality in major trauma, and more than half of delayed deaths are caused by complex immune dysfunction and secondary infections. Systemic inflammatory response syndrome, caused by the release of endogenous factors termed damage-associated molecular patterns (DAMPs) and pathogen-associated molecular patterns (PAMPs) (6, 7), commonly follows traumatic injury. Recognition of DAMPs and PAMPs by the innate immune system triggers both an intense pro-inflammatory and an anti-inflammatory immune response. The anti-inflammatory immune response leads to host defense impairment and sepsis, which increases the risk of multiple organ dysfunction syndrome (MODS) and of death (8, 9). Much money worldwide has been invested in new biological therapeutics for trauma-induced immune dysfunction, but the results are mostly disappointing. The current pro-inflammatory immune paradigm, which is based on an incomplete understanding of the functional integration of the complicated host immune response, remains a major impediment to the establishment of effective, innovative therapies. It is imperative that immunological mechanisms in the pathogenesis of major trauma, particularly the molecular and cellular basis of immune regulation during the early stages of major trauma, are accurately elucidated.

There is growing evidence indicating the existence of a close relationship between autography machinery and immune cells. Autophagy is a crucial catabolic mechanism of non-selective, lysosome-mediated degradation of cytosolic cargo during times of stress. The cytoplasmic cleanup function of autophagy is, by default, anti-inflammatory, in any type of cell capable of activating a cell-autonomous inflammatory response (10, 11). A complementary autophagical function is its involvement in aligning the endoplasmic reticulum (ER) and mitochondrial content with immune cell functions to sculpt the interior of immune cells. In addition, autophagy-dependent metabolic adjustments contribute to immunometabolic states, affecting macrophage and T-cell polarization (12). All of the above findings indicate that different forms of autophagy play key roles in regulating innate and adaptive immunity through affecting inflammatory outputs and resolution (13). A considerable number of studies have been performed to elucidate post-traumatic immune dysfunction over the past few decades, most of which were focused on apoptosis, pyroptosis, depletion, and the generation of immune cells. Few studies have investigated the relationship between autophagy and immunological dysfunction after major trauma. Trauma-induced immunological dysfunction is an extremely complex pathological process, which involves almost all types of immune cells. As autophagy involves many biological processes and signal regulatory pathways, it is difficult to comprehensively analyze its variances and mechanisms using traditional research methods.

At present, little is known about autophagy machinery in immunological regulation after major trauma. To address this issue, we performed single-cell transcriptomics analyses of peripheral blood mononuclear cells (PBMCs) in major trauma patients. The present study aims to explore the transcriptomic profiling of autophagy-related genes in immune cells and to understand how autophagy processes are involved in trauma-induced immune dysfunction. Specifically, the key deregulated biological process and some important hub genes of autophagy involved in immune cells of trauma patients were investigated. Our study indicated that activated autophagy plays a critical role in regulating innate immune responses during the early stages of major trauma. Therefore, our study provides new evidence of autophagy-related mechanisms in the function of innate immune cells after trauma, which may in turn help in the development of new strategies for immune dysfunction prevention and to improve prognoses in the major trauma population.

## 2 Patients and method

### 2.1 Patient information

From April 2021 to February 2022, patients presenting with major trauma and admitted to the SICU (surgical ICU) of Tongji Hospital were eligible for enrollment in this study. Diagnostic criteria for major trauma were based on published guidelines (14, 15). Patients with active malignancy, who were younger than 18 years of age or older than 50 years of age, infected with HIV, receiving immunosuppressive therapy or blood transfusions, and who died within 48 hours of admission were excluded. Patients who had been treated with corticosteroids or other immune regulatory agents before enrollment were also excluded. Finally, five patients, whose characteristics covered a wide range of age and injury severity, were selected for analysis. Control subjects, without significant concomitant acute or chronic illness, were also selected to ensure age and sex comparability in the healthy population. Standard treatments according to published guidelines were provided to all patients (14, 15). The procedures involving human participants were reviewed and approved by the ethics committee at Tongji Hospital and Tongji Medical College. Written informed consent was obtained from patients’ legally authorized representatives or from patients themselves.

### 2.2 Sample collection and isolation of peripheral blood mononuclear cells

Peripheral venous blood samples were obtained within 6 hours of injury and stored under suitable conditions. PBMCs were isolated by density gradient centrifugation using the Ficoll-Paque™ Plus medium (GE Healthcare, Chicago, IL, USA). After centrifugation, the PBMC layer was collected and washed twice in phosphate buffer solution (PBS) at room temperature.

### 2.3 Droplet-based single-cell sequencing

Our small condition RNA (scRNA)-sequencing datasets can be downloaded from the Gene Expression Omnibus (GEO) database (GSE197552). Single-cell RNA sequencing (scRNA-seq) was performed using the Chromium single cell platform (10X Genomics) combined with cell hashing. Approximately 10,000 cells were contained in each channel and 5,000 target cells were recovered. The target cells were lysed and released RNA was barcoded by HTO-barcodes through reverse transcription in individual single-cell gel beads in the emulsion (16). Complementary DNA (cDNA) was generated and amplified following the manufacturer’s protocol, with additional steps for the amplification of HTO barcodes, after which quality was assessed using an Agilent 4200. in accordance with the manufacturer’s instructions, cDNA libraries were sequenced to a depth of 20,000 reads per cell on a Novaseq6000 sequencer (Illumina).

### 2.4 Single-cell RNA-seq data processing

Raw data were aligned to the GRCh38 reference genome using the Cell Ranger v7.0.1 (10X Genomics, Pleasanton, CA, USA) pipeline to generate the unique molecular identifiers (UMIs) count matrices. The output filtered gene expression matrices were analyzed using R software (v.4.0.1 https://www.r-project.org/) with Seurat packages (reference1) (v4.1.1). Low-quality cells were filtered out if they met the following criteria: (1) between <200 and >2,500 unique gene features; (2) between <800 and >10,000 gene counts; and (3) >5% UMIs derived from the mitochondrial genome. The filtered matrix was normalized by employing a global-scaling normalization method (“LogNormalize” in the “NormalizeData” function) and 2,000 highly variable features were identified by the “FindVariableFeatures” function for reducing dimensionality of the datasets. To perform comparative scRNA-seq analysis across experimental conditions, the scRNA-seq integration procedure was commenced by finding the anchors between each cell pair dataset using the “FindIntegrationAnchors” function. After anchors were generated, the integration was performed by using these anchors to generate a comparable scRNA-seq Seurat object (labelled as “integrated”) which contained the integrated gene count matrix of each cell pair dataset. Principal component analysis (PCA) by way of the “RunPCA” function was conducted with default parameters on linear-transformation scaled data generated by the “ScaleData” function. The elbow plot was used to identify the effective number of principal components (PCs) to reflect the difference and the top 10 PCs were chosen for further downstream analyses. Based on the top 10 significant PCs, cells were clustered by FindNeighbors and “FindClusters” function, and we performed t-stochastic neighbor embedding (t-SNE) non-linear dimensional reduction using the “RunTSNE” function with default settings.

### 2.5 Cell type annotation and cluster marker identification

After non-linear dimensional reduction and projection into two-dimensional space by tSNE, cells were clustered together according to similarities in their gene expression profiles. The “FindAllMarker” function with default parameters was used to identify marker genes for each cluster. Cluster annotation was performed based on the canonical markers of particular cell types and excluded clusters expressing two or more canonical cell-type markers.

### 2.6 Autophagy-related gene acquisition

The ATG (autophagy-related genes) genetic toolbox was formed using a well-established methodology designed by Dr. F. Cecconi et al. to monitor autophagy-related genetic transcription (17). A genetic toolbox of 604 autophagy-related genes [including MTOR and upstream pathways (135 genes), autophagy core (197 genes), autophagy regulators (68 genes), mitophagy (80 genes), docking and fusion (22 genes), lysosome (162 genes), and lysosome-related genes (34 genes)] was used to assess the autophagy machinery in cells (17). The ATG genetic toolbox for monitoring autophagy transcription and gene signature enrichment analysis website contains details of genes from these three gene lists: (GOBP_AUTOPHAGY_CELL_DEATH.v.7.5.1, GOBP_REGULATION_OF_AYTOPHAGY_CELL_DEATH.v.7.5.1, and WP_NANOPARTICLE_TRIGGERED_AUTOPHAGIC_CELL_DEATH.v.7.5.1).

### 2.7 Differential gene expression analysis

The standard workflow of differential expression analysis was performed on the bulk RNA gene sets and pseudo-bulk RNA gene sets by using the “DESeq” function in “DESeq2” package (18) (v1.32.1). The log2 fold change (log_2_FC), *p*-values and adjusted *p*-values were extracted using the Result function. Differences in gene expression were considered significant and important if their associated adjusted *p*-value was < 0.05 and they had a |log2 fold change| value of > 1. Volcano plots were created using ggplot2 packages (v3.3.6). As for scRNA-seq differential gene expression analysis, the pseudo-bulk RNA gene matrix was generated according to the aggregation procedure (that is, each individual cell was treated as its own replicate). We then performed differential gene expression analysis, as described above.

### 2.8 Self-organizing map algorithm

Of the 604 genes in the ATG genetic toolbox, 39 genes crucial to processes in autophagy were selected for self-organizing map (SOM) analysis to explore the dynamic expression signature of autophagy, as previously described (17). The expression level of each gene was recorded as the median value for each stage. SOM was constructed by kohonen (v3.0.7) packages and yielded smooth toroidal boundary conditions. The map grid was reset with the top two principal components (PCs) of the data multiplied by the sinusoidal function and visualized by R.

### 2.9 Functional gene enrichment analysis

Gene set enrichment analysis (GSEA) and over-representative analysis (ORA) were performed on the relevant genes using the ClusterProfiler packages (19, 20)(v.4.0.5). Gene ontology (GO) enrichment analysis was performed using the “gseGO” and “enrichGO” functions. Kyoto Encyclopedia of Genes and Genomes (KEGG) enrichment was performed using the “gseKEGG” and “enrichKEGG” functions.

### 2.10 Dataset download and data acquisition

RNA sequencing data from the PBMCs of control participants and severely traumatized patients were downloaded from Gene Expression Omnibus Data Sets (GSE36809, GEO, https://ncbi.nlm.mih.gov/geo/). Peripheral venous blood was sampled from a cohort of 167 severe blunt trauma patients between the ages of 18 and 55 years. Serial blood samples were taken at 12 hours and at 7 and 28 days after injury.

### 2.11 Weighted gene co-expression network analysis

Differentially expressed genes (DEGs) were identified to construct gene co-expression networks using weighted gene co-expression network analysis (WGCNA) packages (21) (v.3.3.4). The co-expression similarity matrix was generated using Pearson correlations and transformed into an adjacency matrix using the soft-thresholding power (β) to ensure a good scale-free topology fit and a large number of connections (the latter was achieved using the “pickSoftThreshold” function). Gene networks were constructed with the condition β = 6 using the “blockwiseModules” function. Autophagy-related modules were identified by detecting the correlation between patient status (control participants and critical trauma patients) and module eigengenes using “cor” functions and the significance of student *p*-values was determined using the “corPvalueStudent” function. The network of most significant modules was constructed by using graph packages (v2.0.5).

### 2.12 Quantification and statistical analysis

For comparison between two independent groups represented as bar plots, the *p*-value was determined with an unpaired two-tailed Student’s *t*-test, with a 95% confidence interval in R. For data in violin plots, a two-tailed Wilcoxon rank-sum test was performed using R. To compare the differential gene expression analysis between bulk transcriptome and pseudo-bulk transcriptome RNA, the false discovery rate (FDR) was determined using R. Data are presented as the mean ± standard deviation if consistent with normal distribution. A value of *p*<0.05 for the differential gene expression analysis indicated statistically significant differences.

## 3 Results

### 3.1 Autophagy: an essential biological process in immune cells’ response to trauma

The abnormal regulation of autophagy in immune processes has been implicated in the development of infectious diseases and cancer (22). However, few studies focus on the impact of autophagy on immune cells after trauma, and little is known about and reported on the precise role of autophagy in trauma-induced immunological dysfunction. The following study aims to demonstrate that autophagy is an essential biological process in immune cells’ response to trauma by way of single-cell transcriptomics analysis.

**Figure 1:**
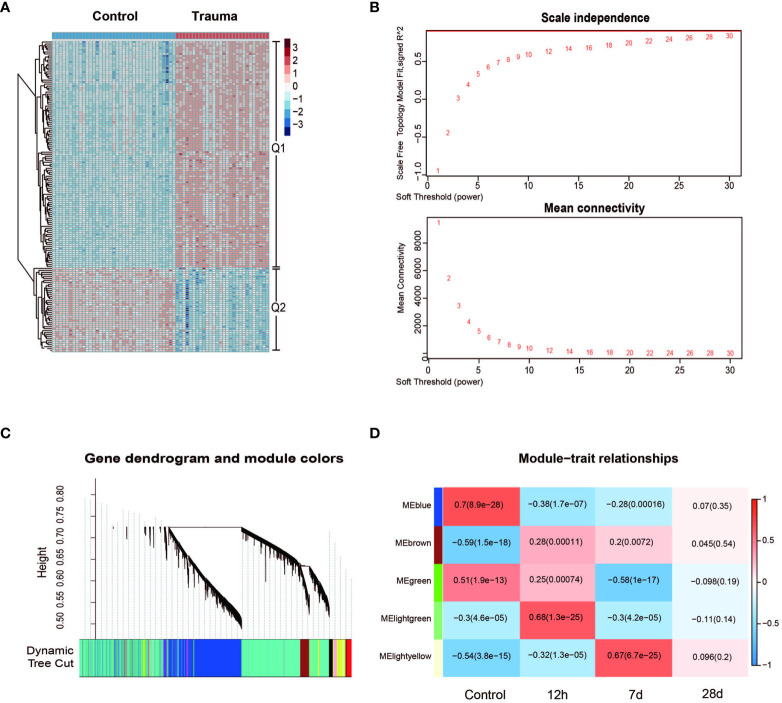
Gene network analysis of trauma patients. **(A)** Heatmap of processed differentially expressed genes (DEGs) expression profiles in trauma patients and control participants. **(B)** Scale independence and mean connectivity under the different soft threshold values. **(C)** Gene co-expression modules, represented by different colors under the gene cluster tree. Each color represents a different co-expression module. **(D)** Heatmap of the correlations between module and the injury time of trauma patients (control, injury after 12 hours, injury after 7 days and injury after 28 days). Five modules that were randomly assigned color labels were identified (blue, brown, green, light green and light yellow). The color of the square area represents the MS (module significance). When the MS value approaches +1, there is a positive correlation between the module and trait; otherwise, when the MS value approaches –1, there is a negative correlation between the module and trait. The *p*-values are shown in parentheses and were calculated using the Student’s *t*-test.

Both gene expression profiles from the GSE36809 dataset and clinical data were downloaded from the GEO database. All transcriptome RNA sequencing data were obtained from PBMCs in a cohort of 167 major trauma patients and control participants. Serial blood samples were taken at 12 hours and at 7 and 28 days after injury. After data filtering, the “DEseq” package in R was used to compare the DEGs in PBMCs for trauma patients with those of control participants. 20,445 DEGs were identified, which are represented visually as both a heatmap (**Figure 1A**) and a dendrogram (**Figure 1C**). A false discovery rate (FDR) of < 0.05 was defined as the threshold for screening DEGs (**Figure 1B**).

**Figure 2:**
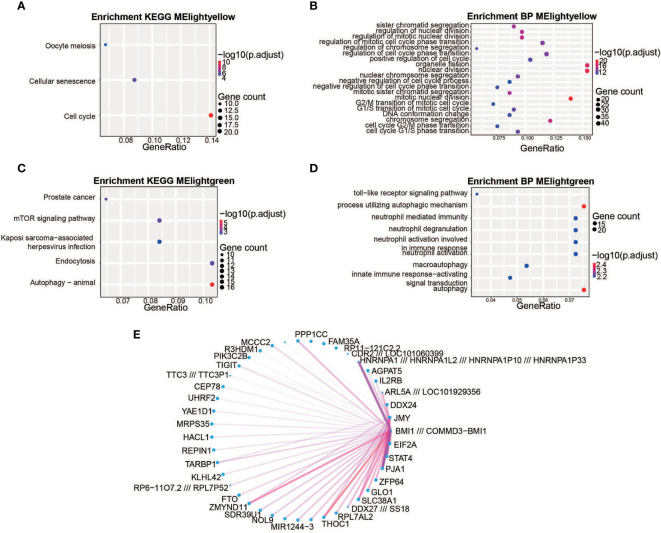
The biological significance of the light-yellow and light-green module genes. **(A, C)** Kyoto Encyclopedia of Genes and Genomes (KEGG) enrichment analysis. The light-yellow module genes were significantly enriched in the cell cycle **(A)** and the light-green module genes were significantly enriched in their autophagy pathways and endocytosis **(C)**. The color represents the -log_10_(adjustment of *p*-value) values and the size of the dot represents the size of corresponding pathways and gene counts. **(B, D)** Gene Ontology (GO) enrichment analysis. The light-yellow module was significantly associated with nuclear division and organelle fission **(B)** and the light-green module was mainly involved in the autophagy process **(D)** The different colors represent the -log_10_(adjustment of *p*-value) values and the size of the dot represents the size of corresponding pathways and gene counts. **(E)** Weighted co-expression gene networks are represented by the light-yellow module. The width of the line represents gene–gene interaction weight and the size of the dot represents the gene degree. JMY is the hub gene for the light-green module.

Weighted gene co-expression network analysis (WGCNA) was performed to categorize DEGs into different gene modules, and 5 gene modules were randomly assigned color labels (**Figure 1D**). The blue module was significantly correlated with DEGs in PBMCs of the control group (r = 0.7, *p* < 0.05), the light-green module was significantly correlated with DEGs in PBMCs taken 12h after injury (r = 0.68, *p* < 0.05), and the light-yellow module was significantly correlated with DEGs in PBMCs taken 7 days after injury (r = 0.67, *p* < 0.05) (**Figure 1D**). Kyoto Encyclopedia of Genes and Genomes (KEGG) analysis was performed on the light-yellow module (7 days after injury) and light-green module (12h after injury). DEGs in the light-yellow module were significantly enriched in the cell cycle (**Figure 2A**), and those in the light-green module genes were significantly enriched in the presence of autophagy pathways and levels of endocytosis (**Figure 2C**). In terms of BP (biological process) enrichment analysis, DEGs in the light-yellow module (7 days after injury) were significantly associated with nuclear division and organelle fission (**Figure 2B**) and those in the light-green module (12h after injury) were mainly involved in the autophagy process (**Figure 2D**). The hub genes networks of the light-green module (12h after injury) showed that JMY (junction mediating and regulatory protein gene) was identified as a hub gene (**Figure 2E**).

### 3.2 Activated autophagy in innate immune cells during the early stages of major trauma

The above-mentioned findings demonstrate that autophagy is an essential biological process in the development of immune disorders in the early stages of major trauma (12h), and subsequent studies have further evaluated the levels of expression profiling of ATG (autophagy-related genes) in innate and adaptive immune cells of patients after major trauma through single-cell transcriptome analysis.

#### 3.2.1 Characteristics of major trauma patients

From April 2021 to February 2022, a total of five patients meeting the study’s eligibility requirements were admitted to the SICU (surgical ICU) of Tongji Hospital. The mean age of the cohort was 37 ± 8.5 years, and all patients were male. The mean Injury Severity Score (ISS) was 28.4± 3.1, indicating a severely injured population, and the mean Glasgow Coma Scale (GCS) score was 11.2 ± 1.3, indicating moderate consciousness disorders. Overall, 40% (2/5) patients suffered from a cerebral injury, 80% (4/5) patients suffered from a chest injury, 40% (2/5) patients suffered from a spine injury, 60% (3/5) patients suffered from pelvic fractures, and 100% (5/5) patients suffered from extremities fractures. Injuries were mainly caused by traffic accidents (*n* = 3), followed by falls (*n* = 1), then crushing (*n* = 1). Peripheral venous blood samples were obtained from three control participants and from five major trauma patients within 6 hours of injury.

#### 3.2.2 Single-cell transcriptional profiling of PBMCs after major trauma

To elucidate the cytologic characteristics of immune cells, scRNA-seq dataset of PBMCs were analyzed as a discovery cohort; this cohort included five patients who had been diagnosed with major trauma. After data filtering, samples with less than 500 cells were excluded. After the quality control process, 167,897 single cells were obtained, with an average of 3,694 unique molecular identifiers (UMIs) and 14,587 genes represented.

**Figure 3:**
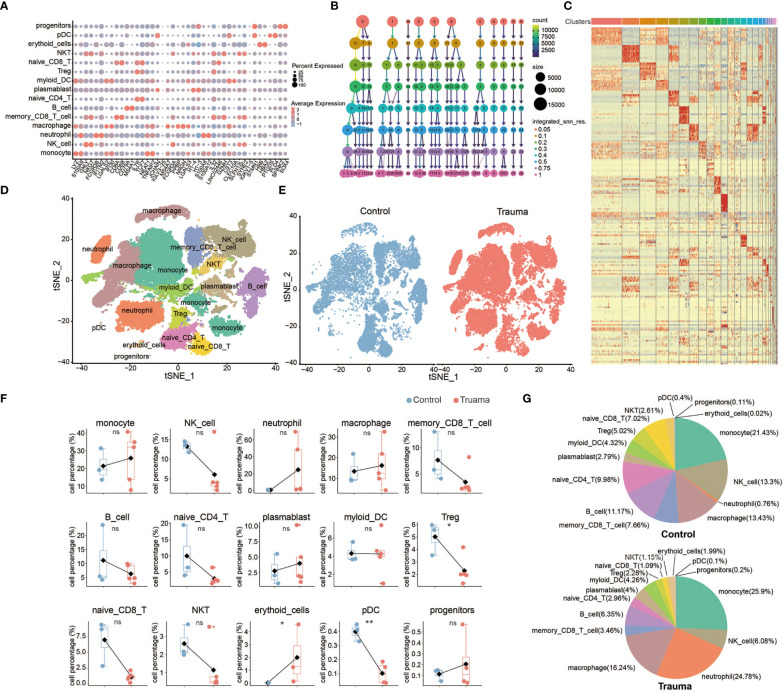
Single-cell transcriptional profiling of peripheral blood mononuclear cells (PBMCs) after major trauma. **(A)** A dot graph showing the expression of hallmark genes by different cell clusters. **(B)** Cluster tree graph of the number of clusters under the conditions of different resolutions. **(C)** Heatmap of the expression of the top 10 hallmark genes by different cell clusters. **(D)** t-distributed stochastic neighbor embedding (t-SNE) of small conditional RNA sequencing (scRNA-seq) of PBMCs from control participants and early-stage major trauma patients. **(E)** t-SNE plot of immune cells in control participants (*n* = 3) and major trauma patients (*n* = 5). **(F)** Quantification of percentage different immune cells from control participants (n=3) and major trauma patients (n=5). *p* values were calculated using an unpaired two-tailed Student’s *t*-test. *p < 0.05, **p < 0.01. **(G)** Proportion of each population out of all immune cells in control participants and major trauma patients. ns, not statistically significant.

Fifteen types of major cells were annotated by expressions of canonical gene markers (23), which included monocytes (LYZ+ and S100A9+), natural killer (NK) cells (GNLY+ and NKG7+), neutrophils (FCGR3B+ and IFITM2+), macrophages (IL7R+ and TCF7+), memory CD8+ *t*-cells (CD8A+, CD8B+ and GNLY+), B cells (MS4A1+ and CD79A), naive CD4+ *t*-cells (CD3+, CD4+ and TCF7+), plasmablasts (PPBP+ and TUBB1+), myeloid dendritic cells (STMN1+ and H4C3+), regulatory *t*-cells (IL7R+ and IL32+), naive CD8+ *t*-cells (CD8B+ and CRR7+), natural killer T (NKT) cells (IL32+, NKG7+ and CD3+), erythroid cells (HBB+ and HBA2+), plasmacytoid dendritic cells (LILRA+), and progenitor cells (SPINK2+ and SOX4+) (**Figures 3A–C**). Fifteen clusters of PBMCs were identified using unsupervised hierarchical clustering and visualized with uniform manifold approximation and projection (UMAP); the cells of each group were then visualized with UMAP plots, individually (**Figures 3D, E**). We further explored the effect of major trauma on the composition of immune cells in PBMCs according to the scRNA-seq data analysis. Only cell types with a number of cells that met the statistical requirement were included in the analysis. As is shown in **Figures 3F, G**, the proportion of NK cells, CD8+ memory *t*-cells, CD4+ naive *t*-cells, CD8+ naive *t*-cells, B cells, Tregs and NKT cells decreased during the early stages of major trauma. The proportion of DCs did not significantly change. Compared with control participants, major trauma patients had significantly increased proportions of monocytes/macrophages and neutrophils (**Figure 3G**).

#### 3.2.3 Expression profiling of autophagy-related genes in immune cells after major trauma

**Figure 4:**
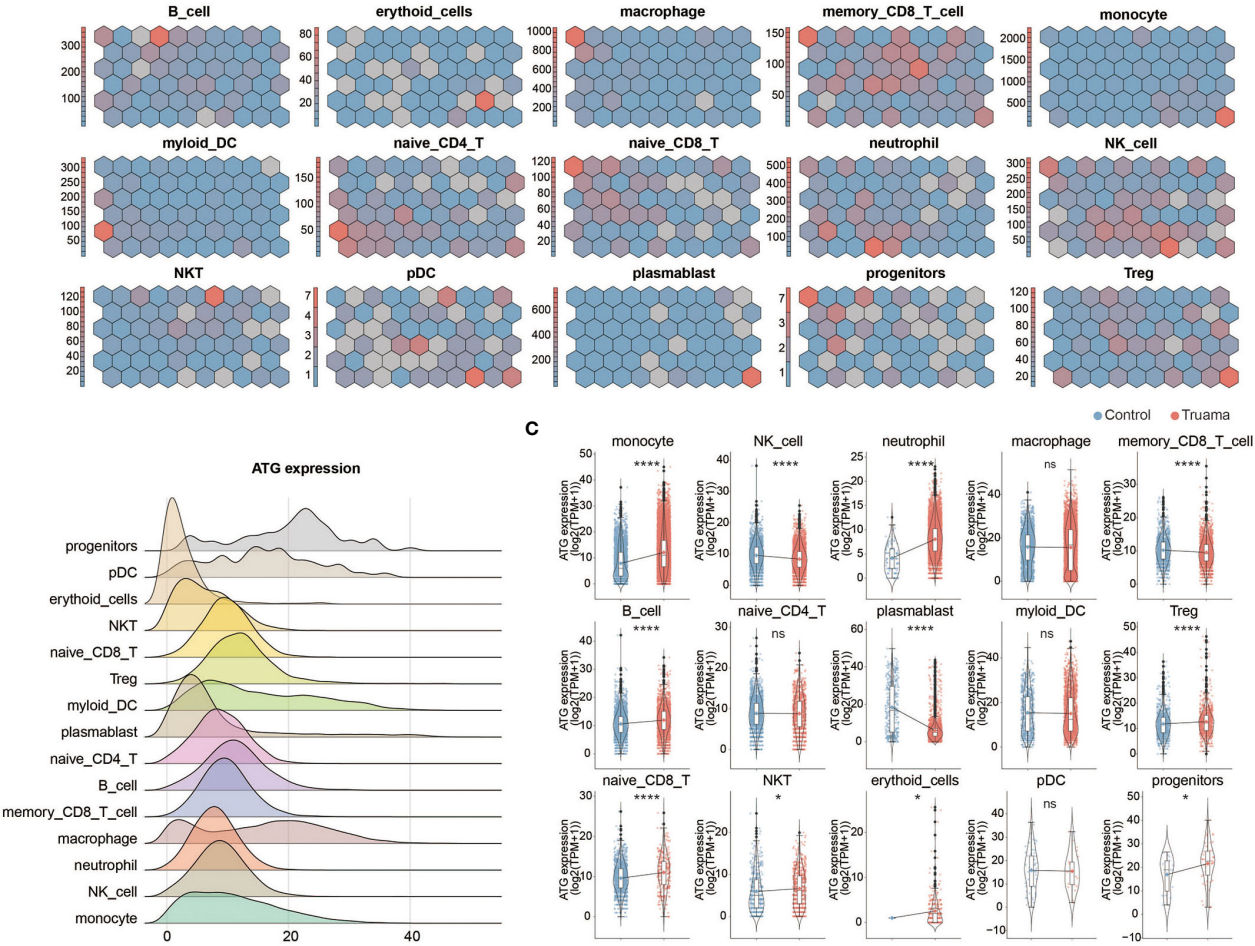
Expression profiling of autophagy-related genes in immune cells after major trauma. **(A)** Expression of autophagic genes represented by the self-organizing map (SOM) algorithm. Each cell cluster represents a subset of autophagy-related genes (ATG). **(B)** Ridges graph showing ATG expression levels in different immune cells. **(C)** The difference in total ATG expression levels of different immune cells between early-stage major trauma patients and control participants. *p* values were calculated using an unpaired two-tailed Student’s *t*-test. *p < 0.05, ****p < 0.0001. ns, not statistically significant.

Given the critical role of autophagy during the early stages of trauma (**Figures 2B, D**), the expression profiling of ATG (autophagy-related genes) was measured in innate and adaptive immune cells of patients within 6 hours of injury. We followed the well-established methodology designed by Dr. F. Cecconi et al. to monitor autophagy-related gene transcription (17). A genetic toolbox with 604 ATG, containing almost all signaling pathways involved in autophagy, was used to assess autophagy in immune cells by single-cell transcriptomics analysis (17). Self-organized mapping (SOM) was performed on each cell type gene expression profile to explore the dynamic transcriptional signature of genes crucial to processes in autophagy (including 39 genes crucial to processes in autophagy) by dimensionality reduction and image analysis. Dynamic expression patterns of genes crucial to processes in autophagy were observed in memory CD8+ *t*-cells, naive CD4+ *t*-cells, neutrophils, NK cells and plasma DC (**Figure 4A**). The total ATG analysis expression analysis outlines the ATG expression profile in each cell type (**Figure 4B**). In the context of trauma patients, the autophagy was significantly activated in innate immune cells, especially in monocytes and neutrophils (**Figure 4C**). No significant change of autophagy level was observed in adaptive immune cells, including memory CD8+ T cells, naive CD4+ T cells, naive CD8+ T cells, Tregs, DCs, and NKT cells.

#### 3.2.4 Activated autophagy in monocytes

**Figure 5:**
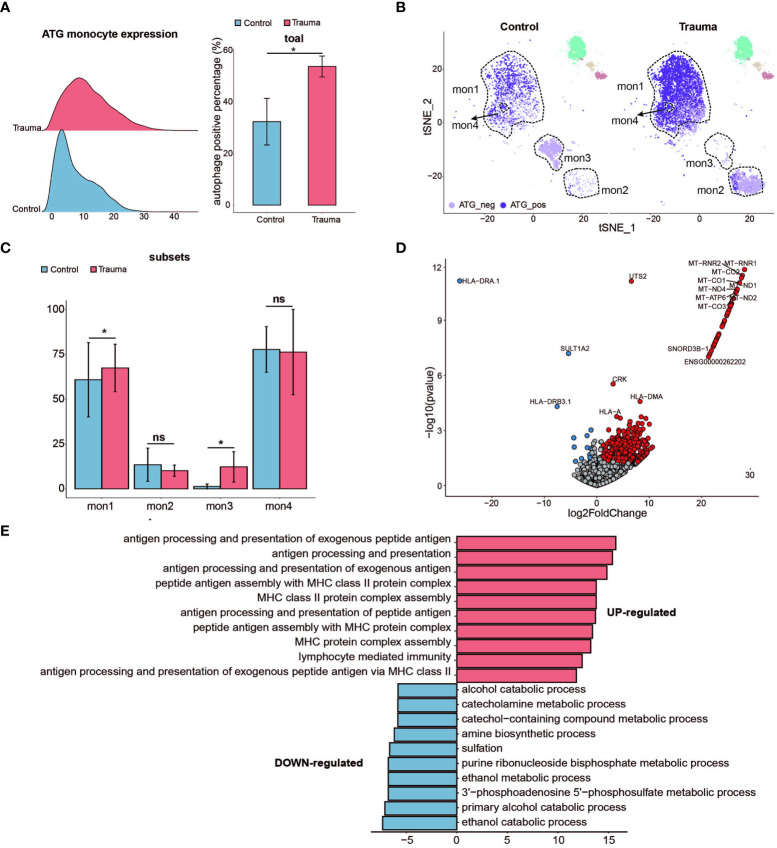
Activated autophagy in monocytes. **(A)** Ridge plot showing the autophagy-related gene (ATG) expression levels of monocytes (left panel) and the percentage of ATG positive monocytes alteration (right panel) in major trauma patients. *p*-values were calculated using an unpaired two-tailed Student’s *t*-test. *p < 0.05. **(B)** t-distributed stochastic neighbor embedding (t-SNE) projection of ATG expression levels of monocytes in major trauma patients. **(C)** The percentage alteration of autophagy-positive monocytes in major trauma patients. *p*-values were calculated using an unpaired two-tailed Student’s *t*-test. **(D)** Volcano graph displaying differential expressed genes of monocytes in major trauma patients and control participants. Genes with log_2_FC values of > 1 and *p*- values of < 0.05 are highlighted in red, and genes with log_2_FC values of < –1 and *p*-values of < 0.05 are highlighted in blue. **(E)** GO analysis of up-regulated differentially expressed genes (DEGs) (red) and down-regulated DEGs (blue) in autophagy-positive monocytes. ns, not statistically significant.

To figure out the influence of the autophagy in monocytes, ATG core gene sets (six core autophagy-related genes, namely *ATG14, ATG7*, *NBR1*, *ULK1, ULK2*, and *WDR45*, were identified as being fundamental to the effective initiation of the autophagy cascade) were used to classify the monocytes (17) (24). The monocytes were divided into two subsets according to the expression of ATG core gene sets, namely ATG positive monocytes (total ATG core genes expression > 10) and ATG negative monocytes (total ATG core genes expression < 10) (24). The percentage of ATG-positive monocytes was greater in major trauma patients than in control participants (**Figure 5A**). Under the condition of resolution=0.05, monocytes were divided into four subsets based on defined stages of cells differentiation, and then the ATG expression levels were determined in each monocyte subset. The quantity of ATG positive monocytes significantly increased in the Mon1 subtype of monocyte after major trauma (**Figures 5B, C**). A total of 645 DEGs were identified in ATG-positive monocytes *via* pseudo-bulk RNA differential gene analysis (**Figure 5D**). Gene ontology enrichment analysis shows that antigen uptake, processing presentation, and MHC class II protein complex assembly pathways were up-regulated in ATG positive monocytes, whereas different types of metabolic processes were down-regulated (**Figure 5E**).

#### 3.2.5 Activated autophagy in neutrophils

**Figure 6:**
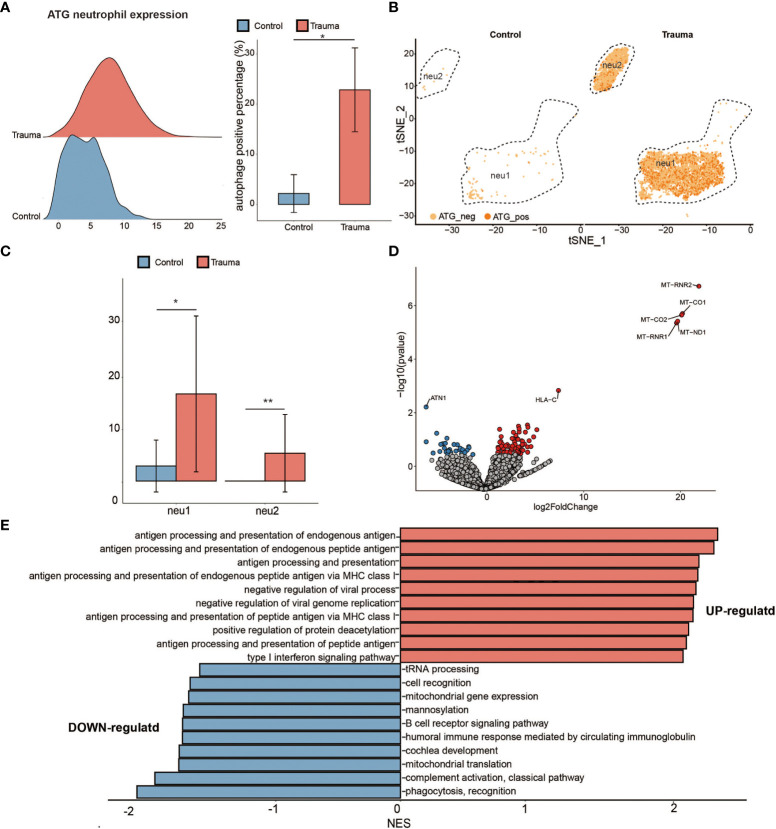
Activated autophagy in neutrophils. **(A)** Ridge plot showing the autophagy-related genes (ATG) expression levels of neutrophils (left panel) and the percentage of ATG-positive neutrophils alteration (right panel) in major trauma patients. *p*-values were calculated using an unpaired two-tailed Student’s *t*-test. *p < 0.05. **(B)** t-distributed stochastic neighbor embedding (t-SNE) projection of ATG expression levels of neutrophils in major trauma patients. **(C)** The percentage alteration of the autophagy positive neutrophils in major trauma patients. *p*-values were calculated using an unpaired two-tailed Student’s *t*-test. **(D)** Volcano graph displaying differentially expressed genes (DEGs) of neutrophils in major trauma patients and control participants. Genes with log_2_FC values of > 1 and *p*-values of < 0.05 are highlighted in red, and genes with log_2_FC values of < –1 and *p*-values of < 0.05 are highlighted in blue. **(E)** GO enrichment analysis of up-regulated DEGs (red) and down-regulated DEGs (blue) in autophagy-positive neutrophils. **p < 0.01.

To explore the function alteration in autophagy positive neutrophils, we separated the neutrophils into ATG-positive and ATG-negative neutrophils according to ATG core gene sets (as mentioned above) (17) (24). The percentage of ATG-positive neutrophils significantly increased in major trauma patients compared with control participants (**Figure 6A**). Neutrophils were divided into two subsets according to cellular differentiation (resolution = 0.05). The quantity of ATG positive neutrophils was upregulated in each of the subtypes individually (**Figures 6B, C**). As shown in **Figure 6D**, 109 DEGs were identified in ATG positive neutrophils *via* pseudo-bulk RNA differential gene analysis (**Figure 6B**). To explore the biological meaning of these DEGs, GO enrichment analysis was performed on DEGs and indicated that there was positive regulation of antigen processing and presentation of endogenous antigen, and that type I interferon signaling pathways were up-regulated in ATG-positive neutrophils. On the contrary, the expression levels of tRNA processing, cell recognition, mitochondrial, phagocytosis and recognition genes were down-regulated (**Figure 6E**).

## 4 Discussion

Hemorrhagic shock and overwhelming injuries of vital organs are responsible for early mortality in major trauma, whereas more than half of delayed deaths are associated with multiple organ failure or severe sepsis induced by the severe repression of immunological functions. Over the past few decades, considerable efforts have been invested on the study of limited pathways or single components in trauma-induced immune dysfunction. However, contradictory findings have been reported, which has always been a major barrier to achieving widely reproducible clinical benefits. Therefore, the underlying molecular mechanism and physiological implications of trauma-induced immunological dysfunction need to be further understood.

In the present study, large-scale single-cell transcriptome analysis and weighted gene co-expression network analysis (WGCNA) provided new insight into the landscape of PBMCs in patients with major trauma. The results of our study demonstrated that the DEGs of the light-green module (12h after injury) were mainly involved in the biological process relating to the autophagy pathways and endocytosis. The Kyoto Encyclopedia of Genes and Genomes (KEGG) pathway analysis and GO enrichment analysis revealed that light-green module (12h after injury) genes were highly enriched in the autophagy pathways, which in turn indicates that autophagy-related genes dominate the expression change in PBMCs during the early stage of major trauma. JMY was screened as the hub gene in light-green modules, and was closely related to the autophagy machinery (25–28). The aforementioned results suggest that autophagy activation is the most significant change occurring in the biological processes of PBMCs during the early stage of major trauma. Previous studies have also observed activated autophagy after trauma, but the majority of findings reach the conclusion that trauma contributes to activated autophagy in osteoblasts, cardiomyocytes or lung tissue using animal models of trauma (29–31). To our knowledge, ours is the first study to investigate autophagy in PBMC after trauma, and to propose that there exists a potential relationship between activated autophagy and trauma-induced immune dysfunction in patients with major trauma.

Autophagy functions broadly in immunity, ranging from cell-autonomous defense to coordination of multicellular immune responses (22). However, there is a lack of study on the role of autophagy in immune cells after major trauma. To simultaneously reveal the autophagy levels in various types of immune cells after trauma, PBMC single-cell sequencing was performed. Single-cell RNA sequencing analysis represents an effective means of obtaining an unbiased and comprehensive visualization of the immunological profiles of PBMCs in patients with major trauma. Compared with bulk RNA sequencing, it has a single-cell level resolution, which can estimate the whole changes of cell subsets, the immune cell function of individual cell, and the correlation between different cell subsets. In using this approach, we observed significantly higher expression levels of autophagy-related gene (ATG) in monocytes and neutrophils accompanying increased cell numbers after major trauma. Identification of ATG genes has provided the impetus for a molecular understanding of autophagy. The findings indicated that autophagy was significantly activated in the innate immune cells of trauma patients. However, almost no significant change in autophagy levels was observed in adaptive immune cells, including naive or memory CD8+ *t*-cells, naive CD4+ *t*-cells, Tregs NKT cells, and B cells. Meanwhile, obviously decreased numbers of adaptive immune cells were observed during the early stages of major trauma. It has been reported that activated autophagy reduced cell damage caused by pathogens, and protected against monocyte death during virus infection (32). Considering the above-mentioned findings, we speculate that alteration in levels of autophagy activation is associated with the function and survival of monocytes after major trauma.

Monocytes possess the potential for differentiation into macrophages and myeloid lineage dendritic cells, which serve three main functions in the immune system: phagocytosis, antigen processing and presentation, and cytokine production (32). There are growing findings which emphasize that aberrations in the function of monocytes are pivotal to the development of trauma-induced immunopathology. Since monocytes bridge innate and adaptive immunity, their dysfunction profoundly affects the whole immune system (33, 34). Activated monocytes and their macrophage and dendritic-cell progeny can directly present antigens to *t*-cells and promote Th1-type immunity. The extremely up-regulated antigen processing and presentation will trigger an intense systemic inflammatory response and excessive release of pro-inflammatory mediators, inevitably leading to unfavorable clinical outcomes in trauma patients. Growing evidence has already confirmed that monocytes pathology contributes to unfavorable clinical outcomes for trauma patients (34, 35). However, few studies reveal the underlying mechanisms behind this.

In the present study, our findings indicated that autophagy was significantly activated in monocytes after major trauma. Previous studies have revealed that the induction of autophagy is essential for the differentiation of monocytes in viral or bacterial infections (32). Autophagy is also important in the induction of pathogen killing by monocytes/macrophages. Preventing the induction of autophagy hinders differentiation and cytokine production in monocytes (32). However, the regulatory effects mediated by autophagy in monocytes are poorly investigated in major trauma patients. In following study, we further elucidated the mechanism of autophagy in regulating monocytes function after major trauma.

GO enrichment analysis further indicated that activated autophagy contributes to the up-regulated major function of monocytes in patients with major trauma, including antigen uptake, processing, presentation, and MHC class II protein complex assembly. Activated autophagy also results in the down-regulation of partial monocytes function, such as ribosome assembly and hydrogen peroxide catabolic process. Our findings indicate that activated autophagy is heavily involved in the pathological changes of monocytes during the early stages of major trauma.

Neutrophils are another important component of the innate immune system and serve as the first line of defense against infiltrating pathogens. There is emerging evidence that the modulated functions of neutrophils play a core role in the development of inflammatory complications after major trauma (36). Hyperactive neutrophils were found to elicit severe inflammatory tissue damage, contribute to develop acute respiratory distress syndrome (ARDS) and multiple organ failure, thus exacerbating outcome after major trauma (37). Over the past decades, the deregulated activation of neutrophils in PBMCs of trauma patients was observed mainly through changes in the phenotypic and intracellular markers of neutrophils in their dynamic response to traumatic insult. At present, little is known about the molecular mechanisms which could potentially regulate neutrophil function and maintain homeostasis in neutrophils after trauma.

As was observed with monocytes, our findings indicated that autophagy was also significantly activated in neutrophils after major trauma. Previous studies have established that autophagy is an important regulator of neutrophil functions, including degranulation, cytokine production and the elimination of invading pathogens in animal models infected with the *Streptococcus* or *Rickettsia* bacterial strains (32). The current evidence around trauma-associated autophagy in neutrophils is inconclusive; however, a correlation between neutrophils and autophagy activation can be observed after major trauma.

In our study, 109 DEGs were identified in ATG-positive neutrophils *via* pseudo-bulk RNA differential gene analysis. To explore the biological significance of these 109 DEGs, GO enrichment analysis was performed. Our findings indicated that activated autophagy contributes to up-regulation in the major function of neutrophils in patients with major trauma, including antigen processing and presentation, and type I interferon signaling pathways. Our findings also indicate that activated autophagy is heavily involved in the pathological changes of monocytes during the early stages of major trauma.

Our study suggests that activated autophagy plays a core role in regulating the pathological processes of innate immune cells during the early stages of major trauma. Activated autophagy causes the hyperaction of innate immune cells, through up-regulating pivotal functions, including antigen uptake, processing, presentation, MHC class II protein complex assembly, and type I interferon signaling pathways. Hyperactive innate immune cells in turn contribute to the development of immunological disorders, which further lead to complex complications such as ARDS, sepsis, MOF, and so on, ultimately exacerbating negative survival outcomes in patients with major trauma.

## 5 Conclusion

In summary, by analyzing single-cell transcriptomics data, our findings suggest the autophagy is significantly activated in monocytes and neutrophils during the early stages of major trauma. Activated autophagy contributes to up-regulated major functions of monocytes and neutrophils. Thus, we contend that well-regulated autophagy activation in neutrophils and monocytes has the potential to optimize post-traumatic immune treatment strategies, which may in turn improve survival outcomes in major trauma patients. Further research investigating the molecular mechanisms of autophagy, and how it affects innate immune cell functions after major trauma need to be conducted in the near future.

## Data availability statement

The datasets presented in this study can be found in online repositories. The names of the repository/repositories and accession number(s) can be found below: https://www.ncbi.nlm.nih.gov/, GSE197522.

## Ethics statement

The studies involving human participants were reviewed and approved by Medical Ethics Committee of Tongji Hospital Affiliated to Tongji Medical College, Huazhong University of Science and Technology. The patients/participants provided written informed consent to participate in this study.

## Author contributions

Z-hT supervised the study. Z-hT and TC designed the experiments, analyzed the data, and co-wrote the manuscript. DC performed the experiments, analyzed the data and co-wrote the manuscript. CZ, HD, JL, SC, PZ, JY, and LD performed the experiments and analyzed the data. All authors contributed to the article and approved the submitted version.
